# *H. Pylori* Treatment in the COVID-19 Era. What Have We Learned So Far?

**DOI:** 10.1007/s11894-024-00922-y

**Published:** 2024-02-02

**Authors:** Konstantinos Ekmektzoglou, Theodore Rokkas

**Affiliations:** School of Medicine, European University Cyprus, 6 Diogenis Str., 2404 Engomi, 22006, Nicosia, 1516 Cyprus

**Keywords:** *H. Pylori*, COVID-19, Treatment, Antibiotics

## Abstract

**Purpose of Review:**

CoronaVirus Disease of 2019 (COVID-19) has negatively influenced the management of multiple conditions in regards to the gastroenterology patient. An equivalent change in the management of *Helicobacter pylori* (*H. pylori*)-related diseases was reported, as practically no eradication treatment was offered during most of the pandemic. Given the scarcity of published data, we performed a literature review trying to elucidate the effect of COVID-19 on *H. pylori* treatment.

**Recent Findings:**

COVID-19 has produced more questions than answers as to the outcome of COVID-19 in *H. Pylori* infected patients, post-COVID-19 patients treated for *H. pylori*, acid suppression and COVID-19 incidence and outcomes, and *H. pylori* eradication treatment in patients having recovered from COVID-19.

**Summary:**

We strongly believe that this scientific uncertainty produced by the COVID-19 pandemic has set up the stage for an incremental change in *H. pylori* treatment as COVID-19 has offered us the chance to speed up how we will, in the near future, approach patients with a possible *Η. pylori* infection.

## Introduction

CoronaVirus Disease of 2019 (COVID-19) has negatively influenced the prevention and management of multiple conditions in regards to the gastroenterology patient as planned outpatient consultations and colorectal cancer (CRC) screening strategies were put on halt for roughly 2 years, mostly due to a concern about the risk (although low) of COVID-19 transmission during gastrointestinal (GI) endoscopy [1]. In the United States of America (USA), endoscopic procedures and inpatient consults were reduced by more than half (53.9% and 51.7%, respectively), while, in the United Kingdom (UK), a 96% reduction in trainee procedures was reported. Similar data were reported from Asia and Australia [2–4]. As things were not complicated enough, numerous studies (mostly retrospective) and meta-analyses confirmed the GI involvement in COVID-19 [5–7].

It is no wonder that an equivalent change in the management of *H. pylori*-related diseases was reported, as practically no eradication treatment was offered for the same time, although a cross-sectional study from China reported that the majority (76.95%) of participants over 40 years old were willing to undergo gastroscopy for early gastric cancer screening, with individuals with known *H. pylori* infection being more likely to undergo gastroscopy [8]. Given that *H. pylori* prevalence estimates are high, with approximately 4.4 billion individuals infected worldwide in 2015 [9], and that higher rates of inflammation, intestinal metaplasia (IM) and *H. pylori* infection were recorded in the COVID-19 period than in the pre-pandemic period [10], researchers have tried to report on associations between *H. pylori* status and treatment and COVID-19.

### What was the Outcome of COVID-19 in *H. Pylori* Infected Patients?

*H. pylori* has been pathophysiologically linked in the pathogenesis of diseases by (a) increasing the expression of angiotensin-converting enzyme-2 (ACE-2) receptors in the GI tract, (b) having a direct association with infection duration and severity, and (c) using its virulent factors to promote immune dysregulation [11]. Even though *H. pylori* infection does not seem to affect the clinical outcome of COVID-19 [12], other researchers have implied that *H. pylori* infection history and IM may increase predisposition for severe acute respiratory syndrome coronavirus 2 (SARS-CoV-2), implying a possible co-pathogenicity [13]; when sucralfate or hydrotalcite was administered in a small cohort of COVID-19 patients with upper GI symptoms, rates of lower GI involvement (diarrhea) were reduced (raising the concept of possible viral load reduction and transmission blockade cranially to caudally via the use of a mucosal protective agent) [14].

However, caution is needed when putting the blame on *H. pylori* as other diseases could partially be held responsible for increasing ACE-2 receptor expression. Furthermore, we have limited knowledge of any symptoms reported before H. pylori or after COVID-19 infection. Many data reported from studies cannot be interpreted safely as a control group of non-COVID-19 patients was missing or studies were significantly underpowered. Last but not least, other non-*H. pylori* factors could be responsible for reported symptoms [15–17].

### Antibiotic Use During the COVID-19 Pandemic

Another aspect that needs to be considered was the use of multiple antibiotics (taken off-label), especially in the early COVID-19 period, for two main reasons. Firstly, and taken into consideration the early necessity for treating a novel disease with increased transmittance and mortality rates accompanied by the scientific obscurity surrounding the nature and mechanism of SARS-CoV-2 infection, repurposing of an already approved drug by regulatory mechanisms, such as the US Food and Drug Administration (FDA) and the European Medical Agency (EMA), was promoted over discovery of new pharmaceutical agents *de novo* due to established drug efficiency and cost-effectiveness. Even though bacterial co-infection was identified in 3.5% and secondary bacterial infection in 15.5% of COVID-19 patients, the end result was the empirical use of broad-spectrum antibiotics in 72% of hospitalized patients in an attempt to treat respiratory symptoms (the same symptoms seen in patients with community-acquired pneumonia) [18–21]. Secondly, the rationale behind prescribing some antibiotics was based on their basic in vitro antiviral and anti-inflammatory/immunomodulatory properties [22, 23].

On one hand azithromycin (a macrolide) was prescribed all too often in the early stages of the COVID-19 pandemic. Macrolide antibiotics interfere with protein synthesis by binding to the 23s ribosomal ribonucleic acid (rRNA) of the 50s ribosomal subunit. However, cross-resistance between azithromycin and clarithromycin (one of the most important antibiotics in current first-line *H. pylori* eradication treatment worldwide) is a well-known fact; the clinically significant mechanism by which *H. pylori* evades clarithromycin is a point mutation in domain V of the 23 S rRNA gene [24, 25]. Meta-analyses do not support the use of azithromycin in the management of COVID-19 [26]. On the other, respiratory fluoroquinolones were recommended in the treatment of community-acquired pneumonia in COVID-19 patients. Levofloxacin is a fluoroquinolone used mainly in the second-line treatment of *H. pylori* eradication. Quinolones inhibit the bacterial type II topoisomerase [deoxyribonucleic acid (DNA) gyrase] and topoisomerase IV, therefore, hindering deoxyribonucleic acid (DNA) synthesis. However, bacterial evasion is also evident after quinolone administration through a mutation of DNA gyrase or topoisomerase IV [22].

### What About Post-COVID-19 Patients Treated for *H. Pylori*?

According to our knowledge, up to now, only one randomized controlled trial (RCT) in Egypt has reported on previously treated as having confirmed or suspected COVID-19 patients with newly diagnosed *H. pylori* infection. A total of 270 naïve *H. pylori-infected* patients with previous treatment for COVID-19 were randomized to either a clarithromycin-based (clarithromycin 500 mg/12 h, amoxicillin 1 gr/12 h, esomeprazole 40 mg/12 h) or a levofloxacin-based regimen (levofloxacin 500 mg/24 h, amoxicillin 1 mg/12 h, esomeprazole 40 mg/12 h) [27]. Although not statistically significant, based on the per-protocol (PP) and intention-to-treat (ITT) analyses, higher treatment response was observed among patients under the levofloxacin-based regimen versus the clarithromycin-based regimen [74.36% and 64.44% (*p* = 0.11) versus 64.66% and 55.56% (*p* = 0.14), respectively]. Interestingly enough, in Egypt, a country with > 50% resistance rates to clarithromycin and no official data on levofloxacin resistance [28], clarithromycin-based triple therapy is still considered a drug of choice to treat *H. pylori* infection, in discordance with the Maastricht VI/Florence consensus report [29, 30]. The authors arbitrarily attributed their even lesser levofloxacin-based regimen eradication rates to an even more increase in levofloxacin resistance in the Egyptian community after the COVID-19 pandemic (aftermath of levofloxacin misuse in the management of COVID-19) [27]. However, one should not forget that both eradication treatments underperformed significantly compared to eradication rates of at least 90% as the desired outcome, thereby rendering their findings of low scientific value [31].

### Acid Suppression and COVID-19 Incidence and Outcomes

Based on the pathophysiological concept that gastric acidity can act as a barrier preventing microorganisms from reaching the intestine [32] and given the true fact that proton pump inhibitors (PPIs) are one of the most common prescribed medicines worldwide [33, 34] associated with increased risk of pneumonia [35], data on PPI use [and other antisecretory drugs like histamine H2 receptor antagonists (H2RA)] and COVID-19 incidence and severity were searched for, both in basic research and in clinical settings [36, 37]. A recent meta-analysis among 18,109 COVID-19 patients under PPIs revealed that PPI use was significantly associated with severe outcomes of COVID-19 [hazard ratio (HR) = 1.53; 95% confidence interval (CI): 1.20–1.95] but not with COVID-19 incidence; H2RA use was significantly associated with decreased COVID-19 incidence (HR = 0.86, 95% CI: 0.76–0.97). Although extreme caution is warranted when trying to interpret studies of substantial heterogeneity and plausible protopathic bias, results seem to favour H2Ras [38]. Based on the aforementioned data, *H. pylori* eradication treatment of a COVID-19-infected patient seems to be justified only if the patient had a previously known peptic ulcer or GI bleeding or before starting anticoagulant treatment but should be put on hold after COVID-19 resolution.

### Pediatric COVID-19 Populations and *H. Pylori*

In accordance with the adult population, elective diagnostic endoscopy in children was substantially suspended (except for emergency cases) during the COVID-19 pandemic [39, 40]. Since the “test and treat” strategy for *H. pylori* infection in children is not warranted but the initial diagnosis of *H. pylori* infection should be limited to invasive gastric biopsy-based methods [41], pediatricians and gastroenterologists failed to identify *H. pylori*-infected children during COVID-19 lockdown. In view of this, experts suggested that diagnosis-wise, in children likely having *H. pylori*-associated gastritis or peptic ulcer disease (based on their presenting symptoms), non-invasive tests [^13^C-urea breath test (UBT) or a stool monoclonal *H. pylori* antigen test], could be employed. Treatment-wise, an empiric eradication strategy could be adopted [42].

### How did *H. Pylori* Eradication Treatment Affect Patients Having Recovered from COVID-19?

Up to now, a few months after the World Health Organization (WHO) declared the end of the 3 years COVID-19 pandemic, with SARS-CoV-2 no longer constituting a public health emergency of international concern (PHEIC) [43], we practically have no concrete knowledge regarding *H. pylori* eradication treatment rates in patients having recovered from a previous COVID-19 infection; no RCT or even preliminary data have been reported.

## Conclusions

While waiting for research to shed some light, editorials and expert opinions highlight a possible future infection control through vaccination control for the benefit of all mankind [44]. Based on the fact that a chronic untreated *H. pylori* infection may induce systemic effects and contribute to tissue damage in distant sites from the stomach, such as the lungs (the respiratory and the digestive tract share a common embryological origin), researchers believe that more efforts are necessary to understand the pathophysiological pathways involved [45]. To make things even more complicated, many epidemiological studies have reported on a possible pathogenetical role of *H. pylori* in extragastric diseases via a potential relation to chronic systemic inflammation and molecular mimicry [46]. Furthermore, the recent context of COVID-19 has renewed the interest of pathologists in diseases of infectious origin [47].

As data on the long COVID-19 syndrome are starting to emerge, we will begin gradually to see the full extent of the long-term GI involvement after COVID-19 resolution [48]. Furthermore, all data suggest that antibiotic resistance has increased over the years of the pandemic (the end result of self-antibiotic and empirical antibiotic medication, and antibiotics prescribed by treating physicians) [49, 50]. New *H. pylori* prevalence studies accompanied by resistance rates with geographical criteria are necessary to establish a new baseline. Furthermore, *H. pylori* societies worldwide should endorse properly designed RCTs, based on non-inferiority methodology, focusing on tailored treatment that should be effective (eradication rates of at least 90%) for the local populations both for the treatment regimen under study as well as for the regimen against which they are compared (the critical comparator is how close the cure rate comes to 100%) [51••,^52^]. Patient-based *H. pylori* therapy does not mean susceptibility-guided treatment alone; patient-based *H. pylori* therapy includes personalized treatment in regards to dosing and duration [31, 53].

If *H. pylori* infection is, by definition, an infectious disease, then it should be governed by the laws of antimicrobial stewardship (as all infectious diseases do) so as to achieve acceptable eradication rates, reduce unnecessary antibiotic prescription and limit the emergence of antibiotic resistance worldwide [52]. The Maastricht VI/Florence consensus report on the management of *H. pylori* infection has already advocated susceptibility testing through polymerase chain reaction (PCR) or next-generation sequencing (NGS) on biological materials (gastric biopsies or stools), recognizing the need for respecting the principles of antibiotic stewardship [30]. The only footnote is whether a molecular-based susceptibility-guided strategy can be used in routine clinical practice. However, antimicrobial susceptibility testing for *H. pylori* is now available and can be used for initial therapy [54, 55]. Profiling *H. pylori* antibiotic resistance by NGS from stool samples provides rapid results highly comparable to those obtained from gastric biopsies [56]. As more and more major diagnostic laboratories in the USA have not only shown interest but now offer practical, rapid, and non-invasive susceptibility testing, through stool PCR or NGS, this may reduce the need for upper GI endoscopies providing, at the same time, reliable antibiotic susceptibility rates. Kits for PCR-based testing for clarithromycin are available and have been approved for clinical use by European regulatory agencies [57]. The respective increase in clinical use will, by causality, lead to price decreases, making them affordable for more and more patients.

**Fig. 1:**
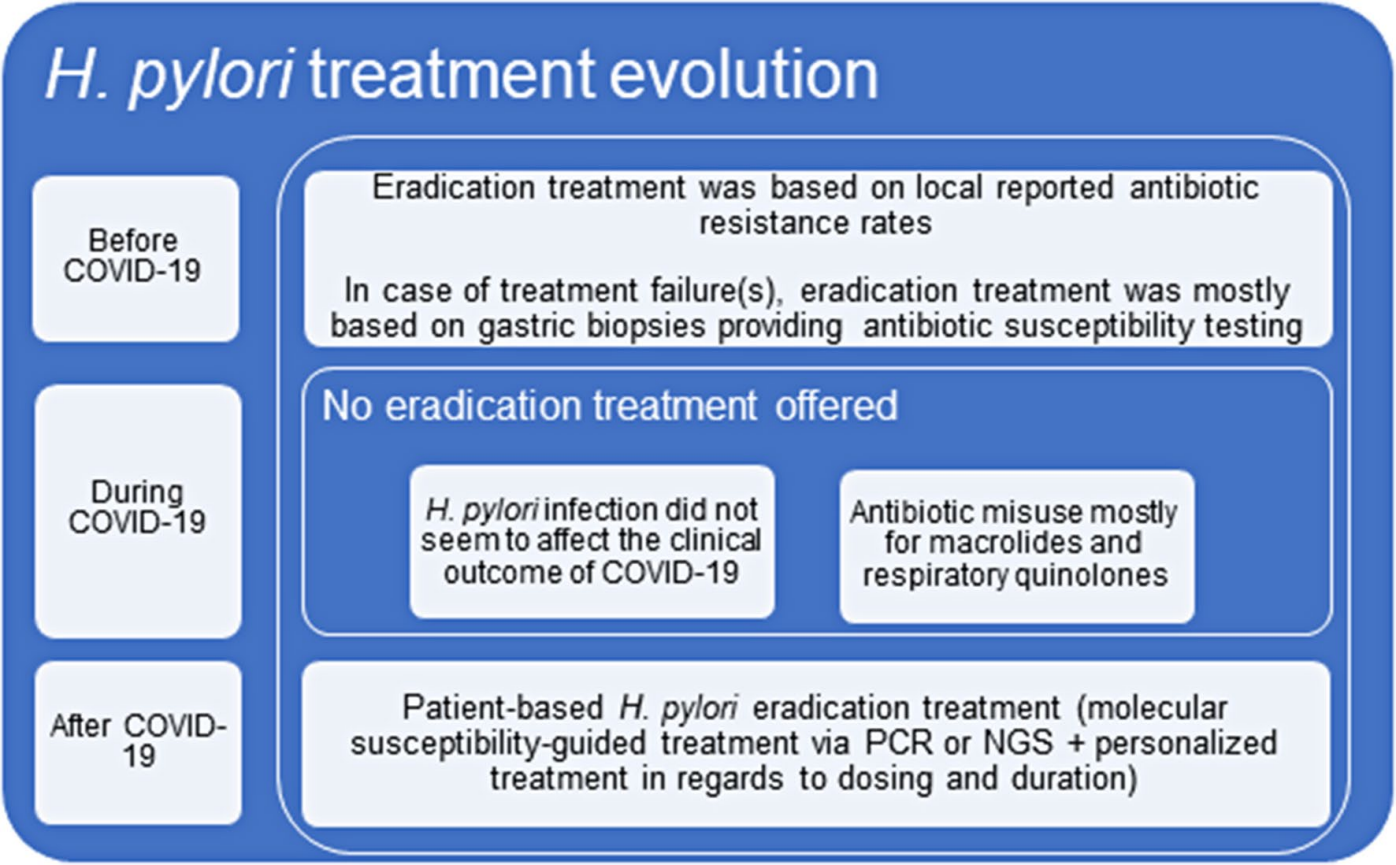
*H. pylori* eradication treatment evolvement over time. COVID-19: CoronaVirus disease of 2019; PCR: polymerase chain reaction; NGS: next-generation sequencing

We strongly believe that the COVID-19 pandemic has set up the stage for such an incremental change in *H. pylori* treatment (Fig. 1). The SARS-CoV-2 infection, amidst its obvious health-related problems, has offered us the chance to speed up, even more, how we will, in the near future, approach patients with a possible *Η. pylori* infection. Antibiotic misuse, common during the COVID-19 era, will no longer pause a threat *to H. pylori* eradication with post-COVID-19 patients (and their treating physicians) not having to worry about worsened local eradication rates or previous antisecretory treatment. Taking into perspective that every cloud (COVID-19) has a silver lining (molecular susceptibility testing), the future for *H. pylori* treatment is now.

## Data Availability

No datasets were generated or analysed during the current study.
